# Supplementary material for: Temperature induced modulation of resonant Raman scattering in bilayer 2H-MoS2
Source: Sci Rep. 2022 Aug 19;12:14169. doi: 10.1038/s41598-022-18439-7 (PMC9391345; doi:10.1038/s41598-022-18439-7)
Supplement: Supplementary file 1 — Supplementary Information. [file 41598_2022_18439_MOESM1_ESM.pdf]

Electronic Supplementary Information:

Temperature induced modulation of resonant Raman scattering in bilayer 2H-MoS_2

Mukul Bhatnagar,[1,*] Tomasz Woźniak,[2] Łucja Kipczak,[1] Natalia Zawadzka,[1] Katarzyna Olkowska-Pucko,[1] Magdalena Grzeszczyk,[1] Jan Pawłowski,[1] Kenji Watanabe,[3] Takashi Taniguchi,[4] Adam Babiński,[1] and Maciej R. Molas[1,†]

[1] Institute of Experimental Physics, Faculty of Physics, University of Warsaw, ul. Pasteura 5, 02-093 Warsaw, Poland

[2] Department of Semiconductor Materials Engineering, Wrocław University of Science and Technology, ul. Wybrzeże Wyspiańskiego 27, 50-370 Wrocław, Poland

[3] Center for Functional Materials, National Institute for Materials Science, 1-1 Namiki, Tsukuba 305-0044, Japan

[4] International Center for Materials Nanoarchitectonics, National Institute for Materials Science, 1-1 Namiki, Tsukuba 305-0044, Japan

* mukul.bhatnagar@fuw.edu.pl

† maciej.molas@fuw.edu.pl

Optical and Atomic Force Microscope images of MoS_2 BL

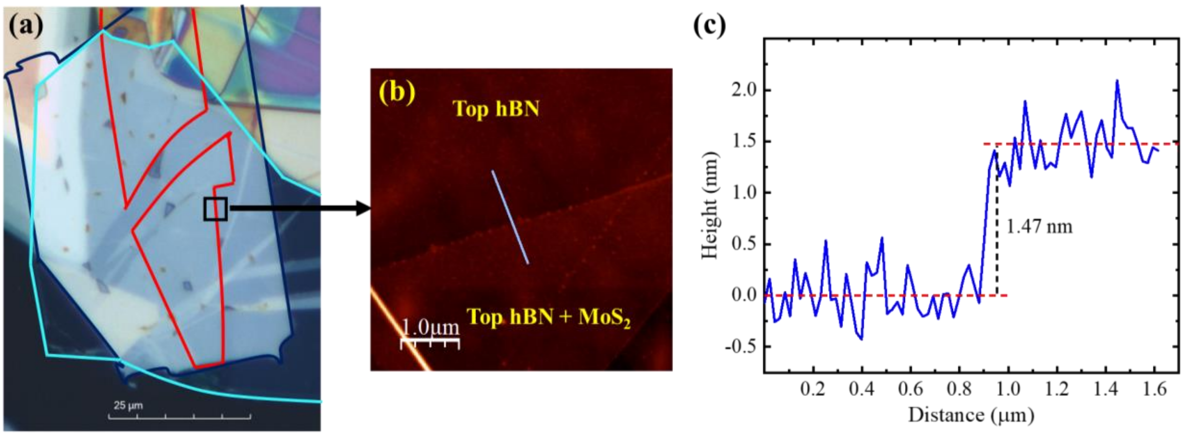

Fig. S1. (a) Optical microscope image of the hBN encapsulated bilayer 2H-MoS_2. The colored boundaries show the bottom hBN (dark blue), bilayer MoS_2 (red), and the top hBN (cyan). (b) False-color atomic force microscope image taken at the respective location marked in (a) by the black square. The blue line represents the height profile drawn in panel (c).

Fig. S1 (a) shows the image from the optical microscope for the investigated MoS_2 bilayer (BL) encapsulated in hBN. It can be seen in the Figure that there are a limited number of bubbles and wrinkles formed during the fabrication process. However, the flat area, bubble-free and wrinkle-free, can be seen, on which the Raman scattering investigations were performed. To confirm the thickness of the studied MoS_2 BL, the atomic force microscopy (AFM) measurement was performed. The false color AFM image is presented in Fig. S1(b). The blue line represents the height profile drawn in Fig.S1(c). The height of 1.47 nm from the line cut across the AFM image is a good fit with previous reports in the literature for bilayer MoS_2 encapsulated in hBN flakes [1]. Compared to the estimation of neutron scattering measurements equal to 0.62 nm [2], the obtained value of 1.47 nm most probably indicates that the equilibrium distance between the hBN and MoS_2 layers differs from that between two neighboring MoS_2 layers in a bulk crystal.

Temperature evolution of the PL spectra measured on MoS_2 BL

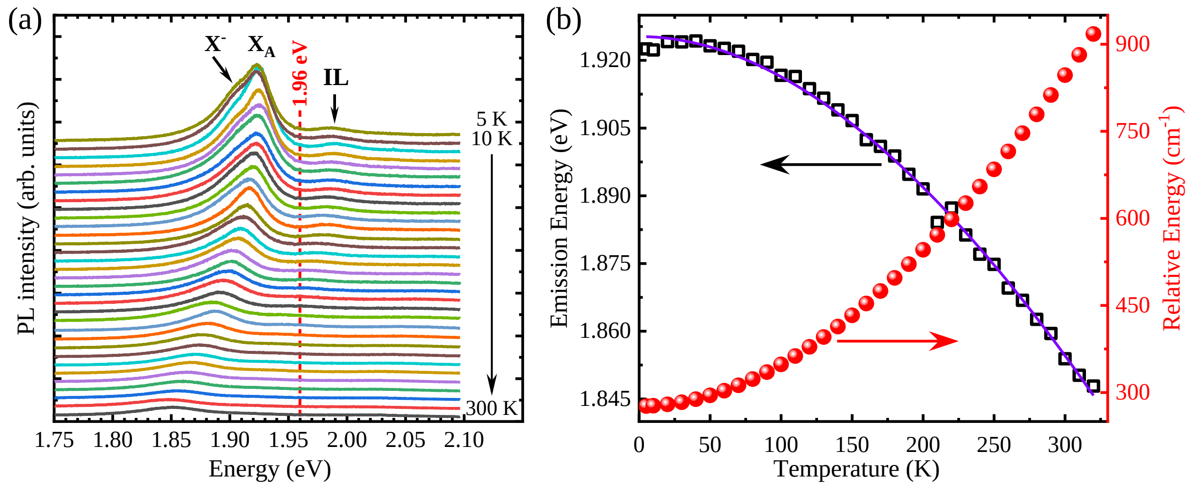

Fig. S2. (a) Temperature evolution of the PL spectra measured on the MoS_2 BL. (b) Open squares show the variation of the emission energy of X_A with change in the applied temperature. The purple curve is fit to the same by Varshni equation. The red spheres represent the calculated relative energy for X_A.

Fig. S2(a) presents the PL spectra measured on the MoS_2 bilayer as a function of temperature, while the temperature dependence of the emission energy of the X_A line accompanied by the fitting of the Varshni equation is shown in Fig. S2(b).

Calculated phonon density of states for BL 2H-MoS_2

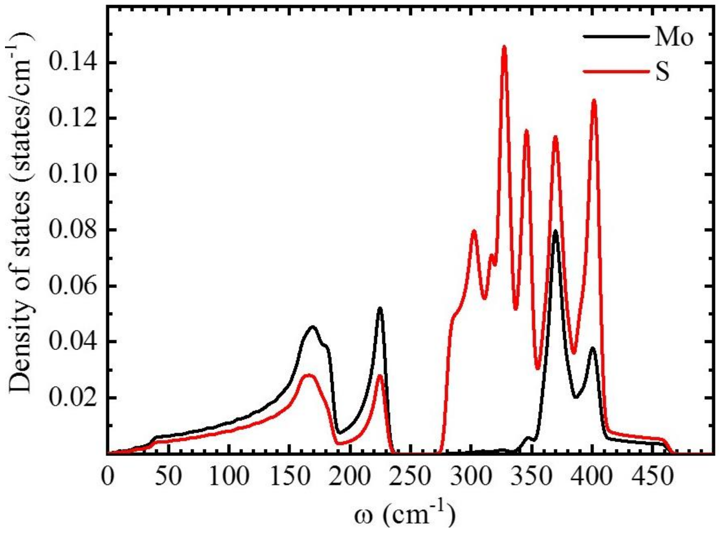

Fig. S3. Phonon density of states for bilayer 2H-MoS_2.

Fig. S3 presents the total density of the phonon states with a defined contribution of molybdenum (Mo) and sulfur (S) atoms to as calculated from first principles. It is clearly observed that the heavier Mo atoms contribute more to the phonon states for frequencies lower than 250 cm^{-1} compared to the heavy S atoms. In contrast, the phonon states with energies larger than 270 cm^{-1} are formed by the S atoms, while the Mo contribution is only limited to the two times smaller energy range.

Temperature dependent Raman spectra of the MoS_2 BL under non-resonant excitation

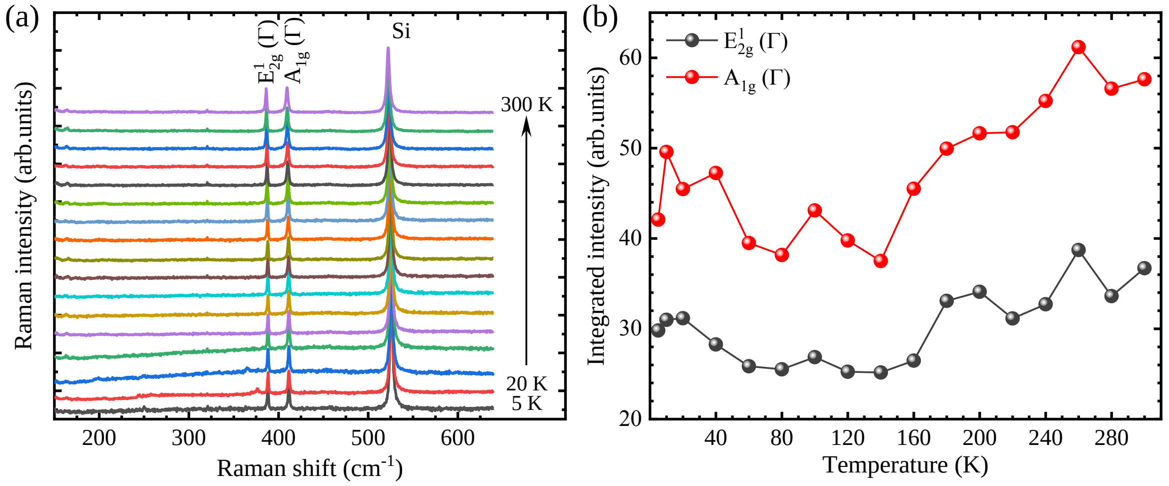

Fig. S4. (a) Temperature dependent Raman spectra acquired under non-resonant condition of excitation where the wavelength of the illuminating light is 515 nm (2.41 eV). (b) Evolution of the integrated intensity with change in temperature for the characteristic E^1_{2g} (Γ) and A_{1g} (Γ) modes.

The Raman spectra, presented in Fig. S4(a), measured under non-resonant conditions for each temperature only shows the characteristic E^1_{2g} (Γ) and A_{1g} (Γ) modes with negligible scattering from

phonon modes at all other energies in the measured range. The extracted integrated intensity for the two modes (see Fig. S4(b)) shows a clear trend where the same increases from low temperature (5 K) to high temperature (300 K), which is in contrast to the intensities of the Raman modes observed under resonant excitation in the main text.

References

[1] A. O. Slobodeniuk, Ł. Bala, M. Koperski, M. R. Molas, P. Kossacki, K. Nogajewski, M. Bartos, K. Watanabe, T. Taniguchi, C. Faugeras, M. Potemski, *2D Materials* **6**, 025026 (2019).
[2] N. Wakabayashi, H. G. Smith, R. M. Nicklow, *Physical Review B* **12**, 659 (1975).
